# Professional staffing in Speech-Language Pathology: challenges and perspectives within the SUS context

**DOI:** 10.1590/2317-1782/e20240275en

**Published:** 2025-08-08

**Authors:** Camila Lima Nascimento, Bruna Gabriela Mechi-Silva, Helenice Yemi Nakamura

**Affiliations:** 1 Departamento de Desenvolvimento Humano e Reabilitação, Faculdade de Ciências Médicas, Universidade Estadual de Campinas – UNICAMP - Campinas (SP), Brasil.

**Keywords:** Public Health, Unified Health System, Health Personnel, Speech, Language and Hearing, Sciences, Workforce

## Abstract

**Purpose:**

The study aims to develop a proposed methodology for workforce planning in Speech-Language Pathology within the Unified Health System (SUS), considering the specific needs of the population and the guidelines from the Ministry of Health.

**Methods:**

An extensive literature review on workforce planning for healthcare professionals was conducted, with a particular focus on Speech-Language Pathology. Based on the collected data, preliminary criteria for staffing parameters were defined, including demand, supply, and current public policies. A flowchart and a preliminary equation for workforce planning were formulated.

**Results:**

Variables essential for workforce planning were identified. Demand was determined by the population with specific needs and the types of services required. Supply was evaluated based on the average working hours of professionals and their productivity. The proposed calculation for workforce planning was based on the demand and supply of professionals and considering five variables: i) population served, ii) population with speech-language pathology-related health needs, iii) distribution of the nature of procedures, iv) average frequency of speech-language pathology follow-ups, and v) average duration of a session. The calculation of supply considers three variables: i) speech-language pathologists, ii) average working hours, iii) dedication to direct patient care.

**Conclusion:**

The discussions on workforce planning is essential to ensure an adequate supply of services to meet the population's needs, improving the organization of care lines and the effectiveness of the services provided.

## INTRODUCTION

The Unified Health System (SUS), the largest public and universal health system in the world, faces important management and planning challenges, considering the complexities and specificities of the Brazilian national territory, which requires, from all health areas, the adequate staffing of the workforce.

This professional staffing is needed in order to ensure the appropriate number and professional skills for each different context and for the health care model embedded in the organization of the system. Therefore, both technical criteria of the population’s health needs and the different jobs in the area have to be considered, in addition to the organization of the different services that compose the health care network (RAS)^(1)^ .

The professional staffing topic has been discussed in the health area, in different categories and with different objectives, but it still needs to be further explored in Speech Therapy, especially with regard to the public service. In order to build an interdisciplinary professional staffing proposal that is appropriate to the specific demands of the population, it is important that there is initially a proposal focused on Speech Therapy that can be used by health managers.

The current distribution of Speech Therapy services and professionals in the SUS presents clear gaps in relation to the population's demand, especially when comparing regions and municipal sizes^(2,3)^.

According to a survey^(4),^ there is a greater concentration of professional employment for speech therapists in the private sector (59%) in Brazil, especially in places where there are more undergraduate courses in Speech Therapy. Data from the Regional Speech Therapy Council of the 2nd region (São Paulo) from 2018 showed a concentration of professional employment in secondary and tertiary care services (96.5%)^(5)^.

The data point to staffing problems in public health services and reinforce the importance of discussing the topic, once inadequacies in professional staffing in Speech Therapy can generate a limiting impact on the professionals' performing options, leading to the fragmentation of healthcare and the weakening of preventive and health promotion actions.

Furthermore, the poor distribution and the insufficient workforce in the area have a negative impact on the care of the population, which do not have access to the vast possibilities offered by the Speech Therapy professional, who can care from newborns to elderly, providing the opportunity to use communication in its various forms. Communication is an essential instrument for the condition of a subject with a recognized place in society and consequent possibility of social engagement.

In order to contribute to this construction, a preliminary staffing calculating proposal was structured, considering studies on professional staffing in health, guidelines from the Ministry of Health (MS) and previous proposals in the Speech Therapy area^(6-8)^. The proposal for an equation applicable to the daily work of health managers aims to develop the first step towards the adequate staffing for speech therapists and entire health teams, considering the organization of health services in the SUS.

## METHOD

This article is an excerpt from the research approved under opinion 3.11.659 by the Research Ethics Committee.

A narrative literature review was conducted to gather available information on professional staffing in different health professions, without period restrictions, aiming to identify the methodologies applied to the staffing of professionals in this area. The materials were read thorough in search for explicit guidelines for staffing calculations.

Based on what was observed in the materials and with the aim of bringing a concrete proposal that could be applied in health services in a simple way, an equation was proposed, developed by the authors, and preliminary criteria were defined for the staffing parameters in the context of Speech Therapy: (a) demand – translated by the projection of the population's health needs; (b) supply – translated by the productivity of the professionals; (c) current public policies.

While the calculation proposal was prepared, the scarcity of epidemiological data and the understanding that each service and territory has specific needs led to the construction of an equation that was simple and flexible. Thus, the relationship between supply and demand was chosen as the framework.

No real-world testing was carried out, being the next step in the development of this work. Furthermore, it is considered, given that this is the first stage, the possibility of developing it into a proposal involving epidemiological studies of the territories and their real demands, in addition to staffing guidelines presented in current public policies involving the profession.

## RESULTS

The details of the preliminary criteria defined, which became the variables used, and the details of the construction of the equation are now presented (Chart 1).

**Chart 1:** Proposal for professional staffing for Speech Therapy

	Variable	Unit	Granularity
Demand (hours/month)	(P_FE_ ) Population served	*# inhabitants*	By territory and by age groups
	(N_FE_ ) Population with health needs related to Speech Therapy	*%*	By age groups
	(D_FE,I_ ) Distribution of nature of procedures	*%*	By age groups and by nature of the procedure
	(f_I_ ) Average frequency of speech therapy monitoring	*Appointments/month*	By nature of the procedure
	(t_I_ ) Average duration of an appointment	*hours/appointment*	By nature of the procedure
Supply (hours/month)	(F) Speech therapists	*# of professionals*	
	(C) Average workload	*hours/month/professional*	Only working hours (holidays, vacations and leaves of absences discounted)
	(D_AT_ ) Dedication to direct service	*%*	Percentage of working hours dedicated to service

### Variables

#### Demand (a)

Demand is determined by the size of the population with health needs related to Speech Therapy and the type of care required, considering the nature of the procedure and the care parameters. It is essential that epidemiological studies be carried out and living conditions in the territories be investigated in order to understand the real needs of the population.

The care parameters for the category are determined by resolution no. 488, of February 18^th^, 2016, of the Federal Council of Speech Therapy (CFFa)^(9)^, based on the definition of time and quantity of procedures per period for a professional, ensuring the provision of quality service.

#### Offer (b)

The provision of speech-language pathology services must take into account the average working hours of the professional and their dedication to direct care for the population, subtracting the time dedicated to planning and administrative activities, in addition to planned and unplanned days off. In order to assess the need for human resources, it is essential to know the current number of professionals employed in health services and their production (number of served users and performed procedures). Therefore, it is of utmost importance that the official public databases of the public health system are fed with updated and reliable information.

#### Public Policies (c)

Current SUS policies that consider the role of speech therapists, for the most part, estimate the need for professionals for each type of service, so it is possible to apply these estimates to the staffing of speech therapists. Some important examples are: the composition of Multiprofessional Teams - eMulti, Home Care - AD, the National Palliative Care Policy (Brazil, 2016/2023/2024)

Policies make up the service provision’s variable, applied according to the service level and the type of recommended action. For example, the speech therapist in the eMulti team works on a broader range, not limited to clinical care, thus bringing a different need for service provision calculation. The professional in Home Care can be part of the multidisciplinary support team (EMAP), in which, accompanied by two other professionals with higher education, the speech therapist will work at least 20 hours. In the composition of the palliative care matrix team (EMCP), the professionals' working hours should be organized to support services and the higher education professionals will be allocated according to local needs and availability.

Despite its importance, the preliminary proposal presented below did not use the guidelines of the policies involving the Speech-Language Pathology professional, with the aim of simplifying the first version of a staffing proposal for the professional category, thus facilitating its use by managers. Based on the inputs generated by the use of this proposal, it will be possible to evolve to the elaboration of a proposal that also contemplates the current policies and the interprofessionalism.

### The proposed calculation of professional staffing

**Figure 1:**
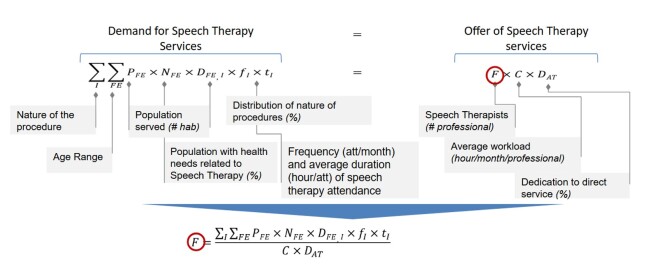
Illustrative equation between the demand and supply relationship criteria of the methodology for the professional staffing proposal in Speech Therapy

The proposal for a professional staffing methodology for Speech Therapy was organized and proposed by the authors of the text, and is based on the document from the World Health Organization (WISN – *Workload Indicators of Staffing Need*)^(10,11)^ which indicates the need for equality between the health demand criteria and the supply of professionals criteria, aiming to guarantee the population's access to services that meet their needs. Thus, the demand calculation includes:

The relationship between these criteria, which is illustrated in the equation detailed in Figure 1 and is based on the need for equality between the health demand criteria and the professional supply criteria, aiming to guarantee the population's access to services that meet their needs. Based on the criteria presented and the proposed relationship between them, it is possible to determine the number of speech therapy professionals needed in a health service, by applying the equation.

## DISCUSSION

The preliminary results of this study show that, based on the variables and the proposed relationship between them, it is possible to determine the number of professionals in a health service by applying an equation, thus providing more concreteness for managers to work with professional staffing for health care services and networks. In order to simplify the proposal, the variables considered encompass the supply and demand criteria - the relationship between the speech therapy needs of the population and the professional contingent -, but it is already stated the need to include aspects that approximate the real local health context from the territory and the population.

For these variables to be truly known, the democratization of health data and information plays a fundamental role. In the field of Speech Therapy, there is still a long way to go in order to achieve broad dissemination and systematization of information on professional capacity and population demands^(12)^.

The biggest challenge is the production of metrics that consider the uniqueness of each location, the diversity of actions and the multiple professions, in addition to the non-standardization of these measures^(13)^. Apart from the workload analysis, other factors need to be considered in the decision-making process, such as structural limitations, specificities of the territory and the size of the restrained demand^(13,14)^.

In this sense, studies on the same topic^(1,13)^ show that, although the use of workload measures related to care demands is more frequent in professional staffing proposals, classifying users according to their clinical conditions and their degree of dependence on care, it is necessary to include more comprehensive variables about the team, the territory, the service network and the population.

Another point to be discussed is the need to balance a formula that is easy to be applied by managers, but that also takes into account the specificities of interprofessional work, such as the non-nuclear work of professionals. Most studies about staffing focus on just one profession, almost exclusively from higher education levels, thus revealing the gap that exists in terms of multiprofessional integration of services.

This difficulty may be linked to the current health care system in Brazil, known by the fragmentation of health actions and services, with a focus on curative actions^(1)^, and which has been insufficient to meet the real needs of the population.

Carrying out professional staffing means acting in technical, technological, scientific and ethical standards, which require a systematization of the assistance to be provided. In this way, it can also contribute to achieving autonomy and professional *status*, which faces challenges related to work, the distribution of professionals and the understanding that professional protagonism is proportional to the understanding that the speech-language pathology community has, regarding its own value^(15)^ .

In this context, it also becomes evident the importance of professional training in health and management, since decision-makers need instruments and a broader understanding of the needs to be addressed in the reorganization of the RAS and in the development of the care lines.

## CONCLUSION

Discussions about healthcare professionals staffing must be deepened so that tools can be debated and offered to provide a range of services that are suitable to the needs of our population, whether through direct assistance or through more effective organization of care lines.

Thus, the proposal presented is a first step towards the actual calculation of professional staffing for Speech Therapy. In order to formulate a robust proposal, it is expected that the next steps will involve testing the use of the formula presented by health unit managers, in order to observe how the calculation behaves, the difficulty of use degree and possible barriers. In addition, it is believed that it is of utmost importance to include variables that contemplate current public health policies, according to the specific RAS service to which the staffing is applied, such as contemplating the policies that involve the implementation of eMulti in case of primary health care services staffing.

The study also highlights the importance of evolving towards an interprofessional staffing proposal that encompasses different professional categories, including higher, technical and secondary levels, for the organization of services within the SUS.

The application of the equation to calculate the staffing has its limitations, such as the scarcity of epidemiological studies in speech-language pathology, which impacts on the knowledge of the specific demands and needs of the population living in the territories where the equation will be applied. Furthermore, it is essential to consider the current number of available professionals and their distribution by level of care and geography, which may compromise the efficiency and access to health and speech therapy services. The use of the tool requires adaptation to the application scenarios, since all the specificities of the territories and services must be taken into consideration. Constant updating is another necessity, since it must follow current public policies, ensuring the alignment with the most recent guidelines and priorities of the health system.

The tool alone does not address the issue of professional staffing in Speech Therapy, but it places itself as a starting point and the direction needed for addressing important health demands.

The proposal presented here comes up as an important first step to assist the decision-making process in the organization of health services, bringing benefits to health professionals in terms of decent work provided by professional staffing, as well as to the population that benefits from higher quality care and qualified access. Finally, professional staffing is a powerful instrument for expanding Speech Therapy within the SUS.
