# Supplementary material for: Professional staffing in Speech-Language Pathology: challenges and perspectives within the SUS context
Source: Codas. 2025 Aug 8;37(4):e20240275. doi: 10.1590/2317-1782/e20240275en (PMC12337711; doi:10.1590/2317-1782/e20240275en)
Supplement: Translation

# Translation: Dimensionamento profissional na Fonoaudiologia: desafios e perspectivas no contexto do SUS

**DOI:** 10.1590/2317-1782/e20240275pt

**Authors:** Camila Lima Nascimento, Bruna Gabriela Mechi-Silva, Helenice Yemi Nakamura

**Affiliations:** 1 Departamento de Desenvolvimento Humano e Reabilitação, Faculdade de Ciências Médicas, Universidade Estadual de Campinas – UNICAMP - Campinas (SP), Brasil.

**Keywords:** Saúde Pública, Sistema Único de Saúde, Pessoal de Saúde, Fonoaudiologia, Recursos Humanos

## Abstract

**Objetivo:**

O estudo visa desenvolver uma proposta de metodologia para o dimensionamento profissional na Fonoaudiologia no Sistema Único de Saúde (SUS), considerando as necessidades específicas da população e as diretrizes do Ministério da Saúde.

**Método:**

Foi realizada uma revisão bibliográfica abrangente sobre o dimensionamento de profissionais de saúde, com especial foco na área. A partir dos dados coletados, foram definidos critérios preliminares para os parâmetros de dimensionamento, incluindo demanda, oferta e políticas públicas vigentes. Foi formulada uma equação preliminar para o dimensionamento na Fonoaudiologia.

**Resultados:**

Foram identificadas variáveis para o dimensionamento profissional. A demanda foi determinada pela população com necessidades específicas e pelos tipos de atendimento requeridos. A oferta foi avaliada com base na carga horária média dos profissionais e sua produtividade. A proposta de cálculo do dimensionamento baseou-se na relação entre a demanda e a oferta de profissionais e considerou cinco variáveis - i) população atendida, ii) população com necessidades de saúde relacionadas à Fonoaudiologia, iii) distribuição de natureza dos procedimentos, iv) frequência média do acompanhamento fonoaudiológico, v) duração média de um atendimento. Já o cálculo da oferta considera três variáveis - i) fonoaudiólogos, ii) carga horária média, iii) dedicação ao atendimento direto.

**Conclusão:**

O aprofundamento das discussões sobre o dimensionamento profissional é essencial para garantir uma oferta de serviços adequada às necessidades da população, melhorando a organização das linhas de cuidado e a efetividade dos serviços prestados.

## INTRODUÇÃO

O Sistema Único de Saúde (SUS), maior sistema de saúde público e universal do mundo, enfrenta desafios importantes de gestão e de planejamento, considerando as complexidades e especificidades do território nacional brasileiro que exige, de todas as áreas da saúde, o dimensionamento adequado da força de trabalho.

O dimensionamento profissional precisa garantir a quantidade e as competências adequadas dos profissionais para cada contexto e para o modelo de atenção à saúde embutido na organização do sistema. Sendo assim, critérios técnicos das necessidades de saúde da população e das diferentes profissões da área precisam ser consideradas, além da organização dos diferentes serviços que compõem a rede de atenção à saúde (RAS)^(1)^.

O tema do dimensionamento profissional vem sendo discutido na área da saúde, em diferentes categorias e com diferentes objetivos, mas ainda carece de aprofundamento na Fonoaudiologia, especialmente no que tange ao serviço público. Para que seja possível a construção de uma proposta de dimensionamento profissional interdisciplinar e adequada às demandas específicas das populações, é importante que inicialmente haja uma proposta voltada para a Fonoaudiologia que possa ser utilizada pelos gestores em saúde.

A distribuição atual dos serviços e profissionais de Fonoaudiologia no SUS apresenta evidentes defasagens frente a demanda da população, especialmente no comparativo entre regiões e portes dos municípios^(2,3)^.

Segundo levantamento realizado^(4)^ há uma maior concentração de vínculos profissionais de fonoaudiólogos no setor privado (59%) no Brasil, especialmente nos locais onde há mais cursos de graduação em Fonoaudiologia. Dados do Conselho Regional de Fonoaudiologia da 2a região (São Paulo) de 2018 evidenciaram uma concentração de vínculos profissionais em serviços de atenção secundária e terciária (96,5%)^(5)^ .

Os dados apontam para problemas de dimensionamento nos serviços públicos de saúde e reforçam a importância da discussão do tema, uma vez que as inadequações no dimensionamento profissional na Fonoaudiologia podem gerar impacto limitante nas possibilidades de ação dos profissionais, levando à fragmentação do cuidado em saúde e o enfraquecimento das ações preventivas e de promoção da saúde.

Além disso, a má distribuição e insuficiência de força de trabalho na área geram impacto negativo no cuidado da população, que não tem acesso às vastas possibilidades ofertadas pelo profissional da Fonoaudiologia, que pode cuidar do recém-nascido ao idoso, oportunizando o uso da comunicação em suas diversas formas. Comunicação que é instrumento essencial para a condição de sujeito com lugar reconhecido na sociedade e consequente possibilidade de participação social.

Com o objetivo de contribuir com essa construção, foi estruturada uma proposta preliminar de cálculo de dimensionamento considerando estudos sobre dimensionamento profissional em saúde, diretrizes do Ministério da Saúde (MS) e propostas anteriores na área da Fonoaudiologia^(6-8)^. A proposta de uma equação aplicável no dia a dia dos gestores de saúde visa evoluir o primeiro passo rumo a um dimensionamento adequado de fonoaudiólogos e de toda a equipe de saúde, considerando a organização dos serviços de saúde no SUS.

## MÉTODO

O presente artigo é um recorte da pesquisa aprovada sob o parecer 3.116.659 pelo Comitê de Ética em Pesquisa.

Foi realizada uma revisão bibliográfica narrativa com levantamento de informações disponíveis sobre dimensionamento profissional em diferentes profissões da saúde, sem restrição de período, com o objetivo de identificar as metodologias aplicadas ao dimensionamento de profissionais na área. Os materiais foram lidos em profundidade na busca por orientações explícitas para o cálculo de dimensionamento.

A partir do que foi observado nos materiais e com o objetivo de trazer uma proposta concreta que pudesse ser aplicada nos serviços de saúde de forma simples, foi proposta uma equação, elaborada pelas autoras e foram definidos critérios preliminares para os parâmetros de dimensionamento no contexto da Fonoaudiologia: (a) demanda – traduzida pela projeção da necessidade de saúde da população; (b) oferta – traduzida pela produtividade dos profissionais; (c) políticas públicas vigentes.

Durante a elaboração da proposta de cálculo, a escassez de dados epidemiológicos e o entendimento de que cada serviço e território tem necessidades específicas levaram à construção de uma equação que fosse simples e flexível. Dessa forma, a relação entre oferta e demanda foi escolhida como estrutura.

Não foi realizado teste em situação real, sendo este o próximo passo para o desenvolvimento deste trabalho. Além disso, considera-se, a partir do entendimento de que essa é a primeira etapa, que seja possível evoluir para uma proposta envolvendo estudos epidemiológicos dos territórios e suas reais demandas, além de diretrizes de dimensionamento apresentadas nas políticas públicas vigentes que envolvem a profissão.

## RESULTADOS

São apresentados agora o detalhamento dos critérios preliminares definidos, que se tornaram as variáveis utilizadas, e o detalhamento da construção da equação. (Quadro 1)

**Quadro 1:** Proposta de dimensionamento profissional para a Fonoaudiologia

	Variável	Unidade	Granularidade
Demanda (horas/mês)	(P_FE_) População atendida	*# habitantes*	Por território e por faixas etárias
	(N_FE_) População com necessidades de saúde relacionadas à Fonoaudiologia	*%*	Por faixas etárias
	(D_FE,I_) Distribuição de natureza dos procedimentos	*%*	Por faixas etárias e por natureza do procedimento
	(f_I_) Frequência média do acompanhamento fonoaudiológico	*Atendimentos/ mês*	Por natureza do procedimento
	(t_I_) Duração média de um atendimento	*horas/ atendimento*	Por natureza do procedimento
Oferta (horas/mês)	(F) Fonoaudiólogos	*# de profissionais*	
	(C) Carga horária média	*horas/mês/ profissional*	Somente horas úteis (já descontados feriados, férias e licenças)
	(D_AT_) Dedicação ao atendimento direto	*%*	Percentual da carga horária dedicado ao atendimento

### Variáveis

#### Demanda (a)

A demanda é determinada pelo tamanho da população com necessidades de saúde relacionadas à Fonoaudiologia e pelo tipo de atendimento necessário, considerando a natureza do procedimento e os parâmetros assistenciais. É essencial que sejam realizados estudos epidemiológicos e investigadas as condições de vida nos territórios para que se compreenda as reais necessidades da população.

Os parâmetros assistenciais para a categoria são determinados pela resolução nº 488, de 18 de fevereiro de 2016 do Conselho Federal de Fonoaudiologia (CFFa)^(9)^, baseados na definição de tempos e quantidade de procedimentos por período para um profissional garantindo a oferta do serviço com qualidade.

#### Oferta (b)

A oferta de serviços fonoaudiológicos deve considerar a carga horária média de trabalho do profissional e sua dedicação ao atendimento direto à população, descontando o tempo dedicado a planejamento e atividades administrativas, além das folgas previstas e não previstas. Para a avaliação da necessidade de recursos humanos é imprescindível conhecer o número atual de profissionais inseridos nos serviços de saúde e sua produção (número de usuários atendidos e de procedimentos realizados). Assim, é de suma importância que os bancos de dados públicos oficiais do sistema público de saúde sejam alimentados com informações atualizadas e confiáveis.

#### Políticas Públicas (c)

As atuais políticas do SUS que consideram a atuação do fonoaudiólogo, em sua maioria, estimam a necessidade de profissionais para cada tipo de serviço, assim é possível aplicar essas estimativas para o dimensionamento dos fonoaudiólogos. Alguns exemplos importantes são: a composição das equipes Multiprofissionais - eMulti, a Atenção Domiciliar - AD, a Política Nacional de Cuidados Paliativos (Brasil, 2016/2023/2024)

As políticas compõem a variável de oferta de serviço, aplicadas de acordo com o nível de serviço e o tipo de atuação preconizada. Por exemplo, o fonoaudiólogo na equipe eMulti atua de forma ampliada, não limitada ao atendimento clínico, trazendo, assim, uma necessidade diferenciada de cálculo de oferta de serviço. O profissional na Atenção Domiciliar, pode fazer parte da equipe multiprofissional de apoio (EMAP), em que acompanhado de mais dois profissionais de nível superior, o fonoaudiólogo, fará no mínimo 20 horas. Na composição da equipe matricial de cuidados paliativos (EMCP), a jornada de trabalho dos profissionais deverá ser organizada para a retaguarda aos serviços e os profissionais de nível superior serão alocados conforme necessidade e disponibilidade local.

Apesar da sua importância, na proposta preliminar apresentada a seguir, não foram utilizadas as diretrizes das políticas que envolvem o profissional de Fonoaudiologia, com o objetivo de simplificar a primeira versão de uma proposta de dimensionamento para a categoria profissional, facilitando assim a utilização pelos gestores. A partir dos insumos gerados pela utilização dessa proposta, será possível evoluir para a elaboração de uma proposta que também contemple as políticas atuais e a interprofissionalidade.

### A proposta de cálculo do dimensionamento profissional

A proposta de metodologia de dimensionamento profissional para a Fonoaudiologia foi organizada e proposta pelas autoras do texto, e é baseada no documento da Organização Mundial de Saúde (WISN – *Workload Indicators of Staffing Need*) ^(10,11)^ que indica a necessidade de igualdade entre os critérios da demanda de saúde e os critérios da oferta de profissionais, com o objetivo de garantir o acesso da população a serviços que atendam às suas necessidades. Assim, o cálculo da demanda compreende:

A relação destes critérios é ilustrada na equação detalhada na Figura 1 e é baseada na necessidade de igualdade entre os critérios da demanda de saúde e os critérios da oferta de profissionais, com o objetivo de garantir o acesso da população a serviços que atendam às suas necessidades. A partir dos critérios apresentados e da relação proposta entre eles, é possível determinar o número de profissionais fonoaudiólogos necessários em um serviço de saúde com a aplicação da equação.

**Figura 1:** Equação ilustrativa da relação entre os critérios de oferta e demanda da metodologia proposta de dimensionamento profissional para a Fonoaudiologia

## DISCUSSÃO

Os resultados preliminares deste estudo mostram que a partir das variáveis e da relação proposta entre elas, é possível determinar o número de profissionais em um serviço de saúde com a aplicação de uma equação, trazendo assim mais concretude para os gestores trabalharem com o dimensionamento profissional para serviços e redes de atenção à saúde. Com o intuito de simplificar a proposta, as variáveis consideradas englobam os critérios de oferta e demanda - a relação entre necessidades fonoaudiológicas da população e o contingente profissional -, mas já é afirmada a necessidade da inclusão de aspectos que se aproximam do real contexto local de saúde do território e da população.

Para que estas variáveis sejam de fato conhecidas, a democratização dos dados e das informações em saúde tem um papel fundamental. No âmbito da Fonoaudiologia, observa-se um caminho ainda a se percorrer a fim de alcançar ampla disseminação e sistematização de informações sobre a capacidade profissional e demandas populacionais^(12)^.

O maior desafio é a produção de métricas que considerem a singularidade de cada local, a diversidade de ações e as múltiplas profissões, além da não padronização dessas medidas^(13)^. Além da análise da carga de trabalho, outros fatores precisam ser considerados no processo de decisão, como limitações estruturais, especificidades do território e tamanho da demanda reprimida^(13,14)^.

Nesse sentido, estudos sobre a mesma temática^(1,13)^ mostram que, embora seja mais frequente nas propostas de dimensionamento profissional a utilização de medidas de carga de trabalho relacionadas às demandas assistenciais, classificando usuários de acordo com suas condições clínicas e seu grau de dependência de cuidados, é necessário incluir variáveis mais abrangentes sobre a equipe, o território, a rede de serviços e a população.

Outro ponto a ser discutido é a necessidade de equilibrar uma fórmula de fácil aplicabilidade pelos gestores, mas que contemple as especificidades da atuação interprofissional, como a atuação não nuclear dos profissionais. A maioria dos estudos sobre dimensionamento focam em apenas uma profissão, quase exclusivamente em formações de nível superior, transparecendo assim a lacuna que há quanto à integração multiprofissional nos serviços.

Essa dificuldade pode estar atrelada ao sistema de atenção à saúde vigente no Brasil, marcado pela fragmentação das ações e dos serviços de saúde, com foco nas ações curativas^(1)^, e que tem sido insuficiente para dar conta das reais necessidades da população.

Realizar o dimensionamento profissional indica atuar em padrões técnicos, tecnológicos, científicos e éticos, que requerem uma sistematização da assistência a ser prestada. Dessa forma, pode contribuir ainda para o alcance da autonomia e do *status* profissional, que enfrenta desafios referentes ao trabalho, à distribuição de profissionais e ao entendimento de que o protagonismo profissional é proporcional à compreensão que a comunidade fonoaudiológica tem a respeito do seu próprio valor^(15)^.

Com esse contexto, fica também evidente a importância da formação profissional em saúde e da formação em gestão, uma vez que os tomadores de decisão precisam de instrumentos e compreensão ampliada das necessidades a serem endereçadas, na reorganização da RAS e na elaboração das linhas de cuidado.

## CONCLUSÃO

As discussões sobre o dimensionamento dos profissionais da saúde devem ser aprofundadas para que sejam debatidas e ofertadas ferramentas que propiciem uma oferta de serviços que seja adequada às necessidades da nossa população, seja pela assistência direta, seja pela organização mais eficaz das linhas de cuidado.

Assim, a proposta apresentada é um primeiro passo rumo ao real cálculo de dimensionamento profissional para Fonoaudiologia. Para que possa ser formulada uma proposta robusta espera-se que os próximos passos envolvam o teste da utilização fórmula apresentada por gestores de unidades de saúde, com a finalidade de observar como o cálculo se comporta, grau de dificuldade do uso e possíveis barreiras. Além disso, acredita-se que é de suma importância a inclusão de variáveis que contemplem as políticas públicas em saúde vigentes, de acordo com o determinado serviço da RAS que o dimensionamento for aplicado, como por exemplo a contemplação das políticas que envolvem a implementação da eMulti no caso do dimensionamento de serviços da atenção primária à saúde.

O estudo aponta ainda a importância de se evoluir para uma proposta de dimensionamento interprofissional contemplando as diferentes categorias profissionais, incluindo nível superior, técnico e médio, para organização dos serviços dentro do SUS.

A aplicação da equação para cálculo do dimensionamento compreende limitações, como a escassez de estudos epidemiológicos em fonoaudiologia, impactando o conhecimento das demandas e necessidades específicas da população residente nos territórios onde a equação será aplicada. Além disso, é essencial considerar o quantitativo atual de profissionais disponíveis e sua distribuição por níveis de atenção e geograficamente, podendo comprometer a eficiência e o acesso aos serviços de saúde e fonoaudiológicos. O uso da ferramenta exige uma adequação aos cenários de aplicação, uma vez que todas as especificidades dos territórios e serviços devem ser levadas em consideração. A atualização constante é outra necessidade, uma vez que deve acompanhar as políticas públicas vigentes, garantindo alinhamento com as diretrizes e prioridades mais recentes do sistema de saúde.

A ferramenta sozinha não endereça a questão do dimensionamento profissional na Fonoaudiologia, mas se posiciona como um ponto de partida e direcionamento necessário para que sejam endereçadas demandas importantes de saúde.

A proposta apresentada nasce como um primeiro passo importante para auxiliar o processo decisório na organização dos serviços de saúde, trazendo benefícios para os profissionais de saúde no que diz respeito aos quesitos de trabalho digno proporcionado pelo dimensionamento profissional, quanto para a população que se beneficia com atendimentos de maior qualidade e com acesso qualificado. Por fim, o dimensionamento profissional apresenta-se como um instrumento potente para a ampliação da Fonoaudiologia dentro do SUS.
